# Effects of Nitrogen Ion Implantation on Wettability and Surface Roughness of WC–Co Tools Used for Wood-Based Panel Machining

**DOI:** 10.3390/ma19061241

**Published:** 2026-03-21

**Authors:** Marek Barlak, Jacek Wilkowski, Radosław Auriga, Jerzy Zagórski, Piotr Boruszewski, Piotr Borysiuk

**Affiliations:** 1 Materials Research Lab, Material Physics Department, National Centre for Nuclear Research Świerk, 7 Sołtana St., 05-400 Otwock, Poland; marek.barlak@ncbj.gov.pl (M.B.); jerzy.zagorski@ncbj.gov.pl (J.Z.); 2 Department of Mechanical Processing of Wood, Institute of Wood Sciences and Furniture, Warsaw University of Life Sciences, 159 Nowoursynowska St., 02-776 Warsaw, Poland; 3 Department of Technology and Entrepreneurship in Wood Industry, Institute of Wood Sciences and Furniture, Warsaw University of Life Sciences, 159 Nowoursynowska St., 02-776 Warsaw, Poland; piotr_bruszewski@sggw.edu.pl (P.B.); piotr_borysiuk@sggw.edu.pl (P.B.)

**Keywords:** wettability, nitrogen ion implantation, WC–Co tools, wood-based panels, machining

## Abstract

**Highlights:**

- WC–Co tools implanted with N ions at 50 kV using fluences of 1 × 10^17^ and 5 × 10^17^ ions·cm^−2^
- SRIM predicts a modified near-surface layer of ~100 nm for both fluences.
- Ra roughness from contact profilometry shows no significant change after implantation.
- Water contact angle drops > 70% at 5 h, indicating markedly higher wettability.
- Wettability partially recovers: contact-angle reduction achieves ~34% after 30 h in air.
- Higher fluence yields consistently lower contact angles, confirming dose dependence.

**Abstract:**

This work explores the effect of nitrogen ion implantation on the wettability of the cemented tungsten carbide–cobalt (WC–Co) tool surface used for wood-based panel machining. Nitrogen ions with an energy of 50 keV and a fluence of 1 × 10^17^ and 5 × 10^17^ cm^−2^ were implanted into the surface layer of commercially available WC–Co indexable knives using the implanter without a mass-separated ion beam. The wettability was characterized by a contact angle instrument. The implantation of nitrogen ions into WC–Co tools caused a statistically significant and practically useful decrease in the contact angle. This obtained effect was dependent on the fluence of the implanted ions, and it changed over time. This effect may also explain the transfer from the workpiece and the surface capture of carbon atoms in the secondary structure formed during the machining of wood materials on tools with ion implantation. On the other hand, the layer of carbon on the surface of the tool formed during machining explains the reduction in friction coefficient observed in experiments and the increase in tool life during cutting.

## 1. Introduction

The wood-based panels industry is a manufacturing industry that secures the work and development of other industries important for the economy, including many small and medium-sized enterprises. Wood-based panels are widely used—the demand for them is constantly growing, and it should be noted that they are ecological products, approximately 90% of which are made of natural raw materials [1,2]. Wood-based panels are practically fully biodegradable, and their material recycling does not pose any major problems. Technologies that recycle used materials into new ones are known and commonly used [3,4]. The machining of wood-based panels requires special tools with increased durability and resistance to abrasive wear. Processing of these materials, used mainly in furniture manufacturing, is a key stage affecting the quality of finished products. The service life of cutting tools is therefore of major importance, as it directly affects the efficiency and cost-effectiveness of wood-based panel machining [5,6,7].

In the machining of various wood-based panels, tools equipped with WC–Co cemented carbide blades are commonly used, as they represent a cost-effective alternative to both more expensive polycrystalline diamond tools and less durable steel tools [8,9]. WC–Co composites consist of hard and brittle tungsten carbide particles embedded in a relatively soft and ductile cobalt binder, which provides a favorable combination of properties, such as high hardness, strength, stiffness, fracture resistance, and wear resistance at temperatures up to 400 °C [10,11,12,13,14,15]. Despite the widespread use of WC–Co composites, the durability of tools made of this type of tool material is still insufficient. Therefore, it is justified to work towards developing methods to improve tool durability.

The service life of WC–Co composite tools can be improved by ion implantation into their surface layer. Ion implantation is a non-equilibrium surface modification technique in which ionized atoms are accelerated in an electric field to velocities ranging from hundreds to thousands of kilometers per second, shaped into a beam, and introduced into the surface of the treated material [16,17]. This method is regarded as a relatively simple, rapid, and cost-effective way of enhancing the tribological performance of machine components and cutting tools [18,19]. The modified area does not form an overlay, so there is no problem of adhesion [20]. There is also no problem of changing the dimensions of the modified element, and, therefore, ion implantation can also be used to modify ready-to-use tools [21,22,23]. Tool life extension can be achieved by reducing the friction coefficient between the tool and the material being processed. For this purpose, elements are introduced into the tested system that show lubricating properties on their own or in combination with others. The most popular are: carbon, boron, phosphorus, chlorine, and sulfur [24]. In addition, recent tribological studies on WC-based systems indicate that appropriately engineered surface modification may significantly affect friction and wear behavior, which further supports the search for improved surface-layer solutions for WC–Co containing cutting materials [25].

It is well known that carbon implanted into the subsurface layer of Al_2_O_3_ ceramics may reduce the friction coefficient and wear of the implanted material [26]. The research results published so far also indicate that implanted boron, in the presence of nitrogen and/or oxygen, can form compounds (BN and B_2_O_3_) showing lubricating properties, which lead to a reduction in the coefficient of friction [27].

Moreover, the lubricating effect of chlorine was investigated, for example, in ceramic TiN layers [28,29], and sulfur in low-alloy 42CrMo_4_ steel [24]. In a tool–workpiece machining system, an additional element is usually introduced into the tools due to their smaller dimensions and weight. However, this is not a rule, because boron can be introduced directly into OSB and MDF boards, which in turn reduces tool wear when processing these types of materials [30].

Ion implantation can also be used to change the surface wettability of different modified materials, both organic materials (e.g., cellulose) [31] and inorganic materials (e.g., steel) [32]. A particularly important parameter correlated with the surface wettability defined by the contact angle is the surface free energy used to assess, for example, the adhesion phenomenon. Adhesion force is associated with the induction of a carbon layer transferred from the workpiece onto the surface of the cutting tool of wood materials. This is called the secondary structure and was created during machining on nitrogen-implanted WC–Co tools. On the other hand, the layer of carbon on the surface of the tool formed during machining explains the reduction in friction coefficient observed in experiments and the increase in tool life during the cutting of wood-based panels [8].

To the best of the authors’ knowledge, the effect of nitrogen ion implantation on the wettability of WC–Co tools intended for wood-based panel machining has not been directly reported so far. The available related studies concern either the surface energy of modified WC–Co elements in bonded steel–carbide systems or wettability-related behavior of WC-based materials under different tribological conditions. Therefore, the present study addresses a more specific and insufficiently explored aspect of WC–Co tool surface behavior.

The aim of the research was to evaluate the influence of nitrogen ion implantation on the surface wettability of WC–Co tools used for wood-based panel machining.

## 2. Materials and Methods

### 2.1. Samples

**Figure 1:**
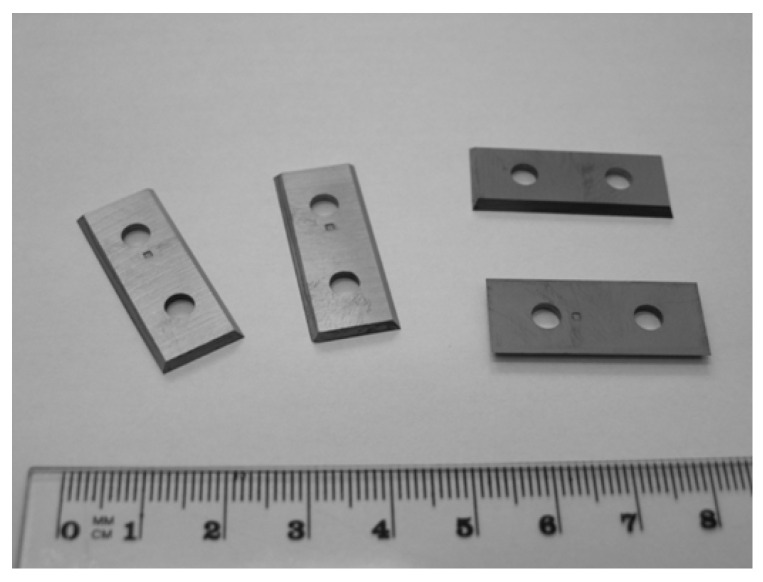
WC–Co indexable knives for wood-based panel machining.

**Figure 2:**
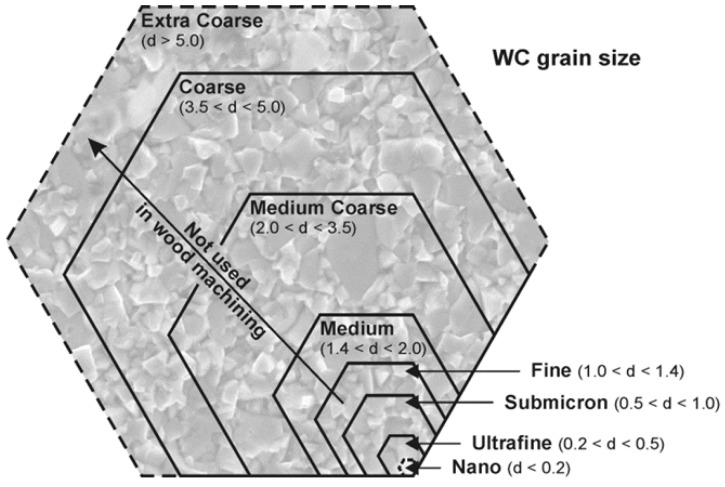
Range of WC grain sizes used in the machining of wood materials.

Commercially available WC–Co tools designed for machining wood-based panels were used in this study. The investigated tools were indexable knives for a milling head, with dimensions of 29.5 mm × 12 mm × 1.5 mm (Figure 1). They were manufactured from a WC–Co composite of the commercial grade KCR08, characterized by a WC grain size of 0.5–0.8 µm, a density of 15.2 g/cm^3^, a hardness of 1885 HV30, and a transverse rupture strength of 2300 MPa. Figure 2 presents a schematic range of WC grain sizes used in the machining of different materials, including wood-based materials. The background of the figure additionally shows an example microstructure of the investigated material obtained at a magnification of 10,000×.

An analysis of commercially available WC–Co grades used for cutting blades showed that composites containing WC grains up to 1 µm and a Co binder content up to 6 wt.% are suitable for machining wood materials. For furniture-grade wood-based panels, manufacturers usually recommend even finer WC microstructures, with grain sizes below 0.8 µm and binder contents lower than 4 wt.%.

The chemical composition of the investigated W–C–Co material was 90.86 wt.% W, 5.94 wt.% C, and 3.20 wt.% Co, which corresponds to 47.4 at.% W, 47.4 at.% C, and 5.2 at.% Co. The cemented carbide grade was supplied by Ceratizit (Reutte, Austria).

### 2.2. Modeling and Ion Implantation

To support the ion implantation procedure, Monte Carlo simulations were carried out for the principal parameters describing the depth distributions of implanted nitrogen, including the maximum dopant concentration (N_max_), projected range (R_p_), range straggling (ΔR_p_), kurtosis, and skewness [33]. The calculations were performed with the freeware package SRIM version 2013.00 by James F.Ziegler (Annapolis, MD, USA) (The Stopping and Range of Ions in Matter) [34]. A total of 100,000 nitrogen ions were simulated for implantation into a W–C–Co target. This substitute composition was adopted instead of WC–Co because the code treats the target as an atomic mixture rather than as a chemically bonded compound. The density used in the calculations was 15.2 g/cm^3^.

Nitrogen was implanted as a mixed beam composed of N_2_^+^ and N^+^ ions in an approximately 1:1 proportion. Under such conditions, two elementary charges correspond to three nitrogen atoms. Consequently, when the accelerating voltage was 50 kV (Table 1), each atom originating from an N_2_^+^ molecular ion carried 25 keV, according to energy conservation. Thus, for a total fluence of 1 × 10^17^ cm^−2^, about 3.3 × 10^16^ cm^−2^ corresponded to 50 keV ions and 6.7 × 10^16^ cm^−2^ to 25 keV ions. The Average Charge State (ACS), commonly used as an equivalent parameter for profile modeling of mixed ion beams, was equal to 0.67 [33].

Implantation was carried out on the clearance faces of WC–Co indexable knives. The ion beam was incident perpendicular to the target surface (0°). The SRIM simulations were performed for absolute zero temperature. Since sputtering of the substrate is not directly accounted for in this model, the theoretical sputtering yield (Y) was additionally estimated using the SUSPRE freeware calculator [35], based on the energy deposited in the surface region according to the Sigmund approach.

The implantation experiments were conducted with a semi-industrial gaseous ion implanter operating with a non-mass-separated ion beam at the National Center for Nuclear Research in Świerk (Otwock, Poland), described in detail elsewhere [36]. Prior to treatment, the knives were cleaned in high-purity acetone.

The base pressure in the vacuum chamber was approximately 8 × 10^−4^ Pa (8 × 10^−6^ mbar). Nitrogen with a purity of 5 N served as the working gas. Two implantation fluences were applied: 1 × 10^17^ cm^−2^ and 5 × 10^17^ cm^−2^. The accelerating voltage was set to 50 kV. This value was selected as a practically justified treatment condition, consistent both with typical energy ranges used in semi-industrial ion implantation systems and with our previous investigations on WC–Co tools. Higher nominal energies were not considered here because they would move the implanted zone deeper into the substrate, whereas the property analyzed in the present study—surface wettability—is governed primarily by the near-surface region. Likewise, fluences on the order of 1 × 10^17^ cm^−2^ are standard in tribological ion implantation studies and had already been validated in our earlier work. Therefore, the present study was intended as an evaluation of wettability for selected implantation conditions rather than as an optimization of beam energy or dose.

The continuous beam current was about 300 µA, corresponding to an ion current density of approximately 15 µA/cm^2^ for an implantation area of 20 cm^2^. The beam diameter was about 50 mm and did not show a significant reduction in current density across the working area. Because the beam fully covered the mounted tools, scanning was not required, and the samples were positioned in the central part of the beam. During implantation, the temperature was monitored by a thermocouple attached to the sample holder rather than directly to the tools; therefore, the reported tool temperature should be regarded as an estimate. Under these conditions, the maximum temperature was assumed not to exceed 200 °C. The treatment time was 12 min for the lower fluence and 60 min for the higher fluence. Overall, the adopted implantation parameters were in agreement with our earlier studies [37,38].

### 2.3. Surface Roughness Measurement

Surface roughness of WC–Co blades was measured using a Mitutoyo SJ-201 contact profilometer (Mitutoyo Corporation, Tokyo, Japan) equipped with a diamond stylus in the form of a spherically tipped cone (tip radius 2 µm, cone angle 60°). Measurements were performed with a sampling length of 0.25 mm and an evaluation length of 1.25 mm (5 × 0.25 mm). A Gaussian low-pass filter was used to limit the influence of random errors.

**Figure 3:**
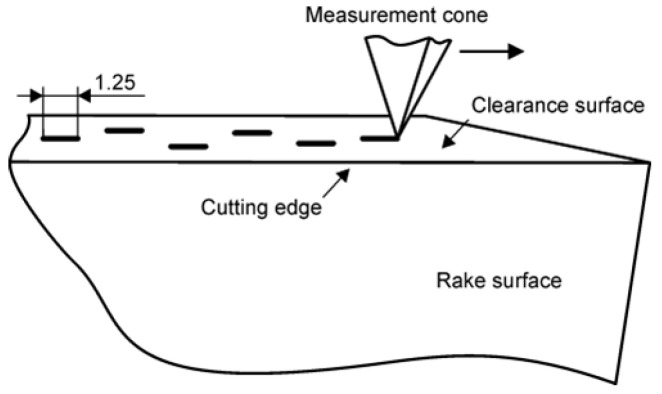
Scheme of measuring the roughness of the clearance surface.

Profilometer traces were recorded on the clearance surface, parallel to the main cutting edge. Six repetitions of the measurements were performed for each type of sample (Figure 3). Roughness parameters R_a_ were calculated from the recorded profiles in accordance with ISO 4287 [39] and ISO 4288 [40].

### 2.4. Wettability Tests

Virgin and nitrogen-implanted WC–Co tools were evaluated in terms of wettability by measuring the water contact angle using a Phoenix 300 goniometer (Surface Electro Optics, Suwon City, Republic of Korea) in the sessile-drop configuration. Droplets of distilled water with a volume of 0.2 µL were dispensed onto the tool surface. The measurements were performed under controlled ambient conditions (T = 20 °C, RH = 65%). The contact angle was determined using an image analysis system (Phoenix 300 Surface Electro Optics, Suwon City, Republic of Korea) as the angle between the tangent to the droplet contour at the three-phase contact point and the baseline.

Dynamic wetting behavior was recorded 5, 10, 20, 30, 40, 50, and 60 s after droplet deposition. Wettability tests were carried out 5 h and 30 h after completion of the ion implantation process. The tool surfaces were not additionally cleaned prior to the measurements. For each experimental variant, six droplets were analyzed, and the reported values represent the mean of these replicates. Between implantation and the wettability measurements, the tools were stored under ambient conditions (20 ± 2 °C, 65 ± 5% air humidity).

### 2.5. Statistical Analysis

The experimental data were statistically analyzed using STATISTICA 13.3 software (TIBCO Software Inc., Palo Alto, CA, USA). The determination of each property was carried out for at least six replicates for a given variant of the material. In order to compare the measured values, statistical tests were used to verify one-dimensional properties such as variance and arithmetic mean, i.e., Snedecor’s F-test and Student’s *t*-test. The significance level was assumed to be 0.05.

## 3. Results and Discussion

### 3.1. Results of Modeling Nitrogen Ion Implantation

**Figure 4:**
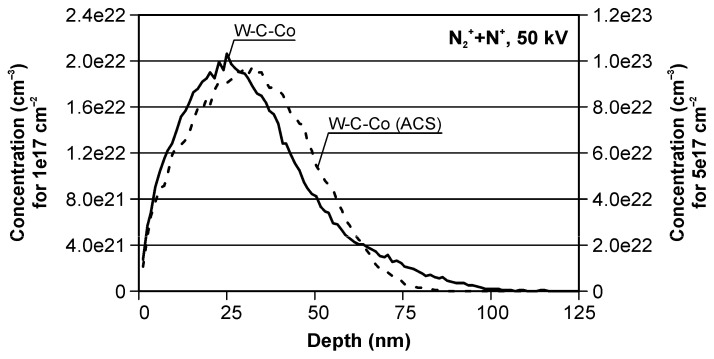
Modeled nitrogen depth profiles in WC–Co.

Figure 4 shows the SRIM-modeled nitrogen concentration–depth profiles in the WC–Co substrate implanted with a mixed nitrogen beam containing N_2_^+^ and N^+^ ions, together with the corresponding Average Charge State (ACS) used as an effective description of the mixed charge-state distribution. The simulations were performed for a total fluence of 1 × 10^17^ cm^−2^ and 5 × 10^17^ cm^−2^ at an accelerating voltage of 50 kV. SRIM is a widely adopted BCA Monte Carlo code for first-order prediction of implantation ranges and concentration profiles, particularly useful for comparing processing variants at fixed energy and target composition [41].

For both fluences, the modeled profiles indicate that the modified zone is confined to the near-surface region. Although the projected range Rp is on the order of ~32 nm (Table 2), the distribution tail extends deeper due to straggling; therefore, the effective affected thickness (e.g., ~*R_p_* + 2Δ*R_p_*) reaches approximately ~0.1 µm, consistent with the ~100 nm depth scale visible in Figure 4. This near-surface confinement is typical for low-energy nitrogen implantation and is relevant for cutting tools where wear and adhesion phenomena are governed primarily by the topmost tens to hundreds of nanometers [37,42].

As expected for static SRIM profiles, increasing the implanted fluence by a factor of five leads to an approximately five-fold increase in the peak dopant concentration *N_max_*, while the characteristic depth parameters remain nearly unchanged (Figure 4, Table 2). This behavior follows directly from the fact that, at constant energy and target composition, the range statistics (*R_p_*, Δ*R_p_*) are mainly controlled by collision kinematics, whereas fluence scales the number of implanted atoms [34,41].

Table 2 summarizes the peak concentration and the statistical descriptors of the modeled nitrogen distributions (*R_p_*, Δ*R_p_*, skewness, and kurtosis) as well as the sputtering yield. The results show that *N_max_*, *R_p_*, Δ*R_p_*, and *Y* are very similar for the explicit (N_2_^+^ + N^+^) case and the ACS-equivalent approximation. Hence, the ACS description can be used as a practical surrogate for estimating the first-order profile parameters and sputtering yield under the studied conditions (50 kV, WC–Co target), which simplifies calculations for mixed beams typical of non-mass-separated implanters.

In contrast, skewness and kurtosis differ substantially between the explicit mixed-beam calculation and the ACS-equivalent profile. This discrepancy is expected because the mixed beam represents a superposition of at least two implantation sub-profiles (with different energy-per-atom), and the higher-order moments are sensitive to asymmetry and tail contributions. Therefore, while ACS equivalence is adequate for R_p_/ΔR_p_-level comparisons, it is not recommended for analyzing fine profile-shape descriptors (skewness/kurtosis), particularly if these descriptors are later correlated with experimentally measured depth profiles or near-surface chemistry. Similar caution is commonly noted when comparing static SRIM predictions to cases where multi-species beams and/or dynamic surface evolution become relevant [35,36,43,44].

Finally, the near-surface localization predicted here is consistent with prior reports where nitrogen implantation of WC–Co leads to measurable changes in tribological response and tool performance, attributed to modifications within a shallow surface layer (including formation of carbon-enriched layers and/or nitride-related phases depending on conditions) [9,37,38,42].

### 3.2. Surface Roughness Analysis

**Figure 5:**
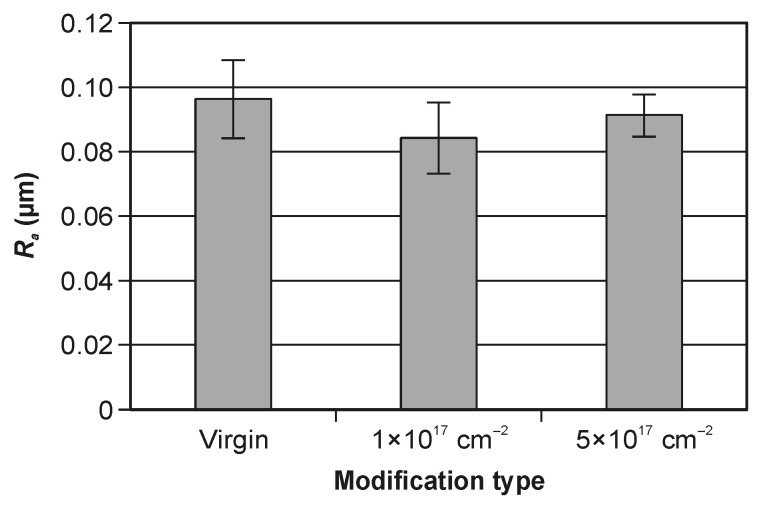
Clearance surface roughness of the WC–Co indexable knives for modification types.

Figure 5 presents the arithmetic mean roughness R_a_ measured on the clearance face of WC–Co indexable knives (Figure 3) for the virgin tools and for the tools modified by nitrogen ion implantation. The bar chart shows mean values with 95% confidence intervals (α = 0.05). Under the applied conditions, no statistically significant differences in mean R_a_ were observed between the investigated groups. This result is consistent with the nature of ion implantation, which primarily affects the near-surface chemical composition and subsurface zone rather than the micrometric surface topography detectable by stylus profilometry. Therefore, within the sensitivity of stylus profilometry and for the R_a_ descriptor, nitrogen implantation did not measurably alter the macroscopic geometrical structure of the clearance surface [45].

This outcome is physically plausible because ion implantation at the applied energy primarily modifies a near-surface zone (tens of nanometers to ~0.1 μm) and usually does not induce large-scale topographical changes unless significant sputtering, cracking, or thermally driven surface damage occurs. In other systems, roughness changes after nitrogen implantation have been attributed to stress-assisted microcracking or irradiation-induced surface damage; however, the presence and magnitude of such effects depend strongly on the substrate, implantation parameters, and temperature [46].

In addition, contact stylus measurements are influenced by measurement settings (e.g., tracing speed and filtering) and have limited sensitivity to nanoscale modifications, so ion-induced changes confined to the near-surface region may remain undetected if they do not translate into micrometer-scale profile alterations [47,48].

Consequently, the absence of statistically significant differences in R_a_ suggests that any implantation-driven changes discussed in subsequent sections (e.g., wettability or tool performance) are more likely associated with near-surface chemical/microstructural modification rather than with a change in the overall roughness amplitude. To strengthen this conclusion, future analysis could complement R_a_ with selected areal texture parameters (ISO 4287) and/or higher-resolution topography methods (optical profilometry/AFM) to capture potential nanoscale effects [47].

### 3.3. Wettability Analysis

**Figure 6:**
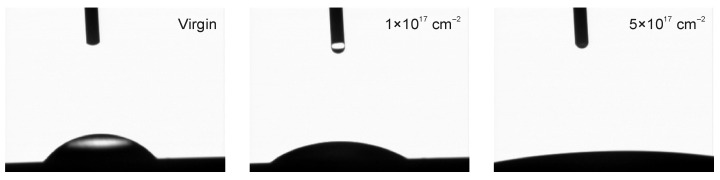
Representative droplets used for contact angle measurements on the WC–Co surface.

**Figure 7:**
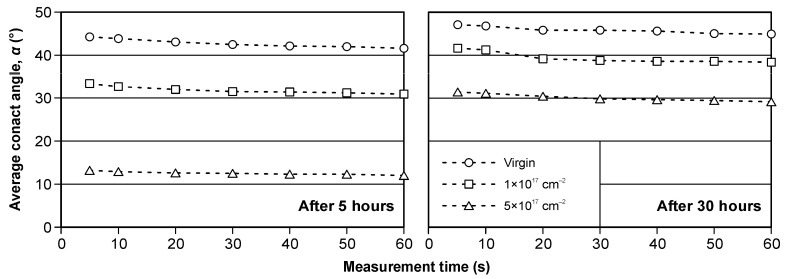
Changes in contact angle as a function of time, measured 5 h (**left**) and 30 h (**right**) after implantation. Data are presented as mean values (*n* = X) with 95% confidence intervals (α = 0.05).

Figure 6 shows representative water droplets recorded during sessile-drop contact angle measurements on WC–Co surfaces. Clear differences in droplet shape are observed between the virgin and ion-implanted variants, indicating implantation-induced changes in surface wettability. Figure 7 summarizes the time evolution of the water contact angle (5–60 s after droplet deposition) measured at 5 h and 30 h after the implantation process. In both datasets, the virgin surface exhibits the highest contact angle (lowest wettability), whereas increasing nitrogen fluence leads to a systematic decrease in the contact angle, i.e., enhanced wettability.

A reduction in contact angle after ion implantation has been reported for various material classes and is commonly attributed to an increase in the polar component of surface energy caused by implantation-induced chemical modification and/or removal of weakly bound adventitious contamination during energetic ion exposure [49,50].

**Figure 8:**
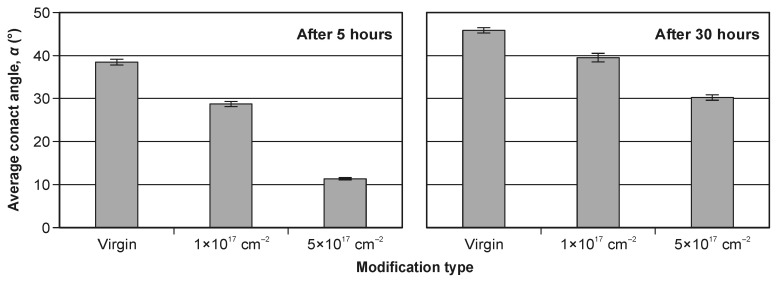
The average contact angle for modification types—5 h (**left**) and 30 h (**right**) after implantation.

**Figure 9:**
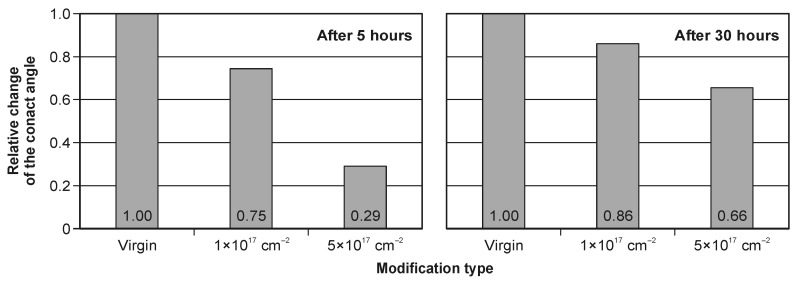
Relative change in the contact angle for modification types—5 h (**left**) and 30 h (**right**) after implantation.

The contact angle measured 30 h after implantation is higher for all variants compared to the 5 h measurements (Figure 7, Figure 8 and Figure 9), indicating time-dependent wettability decay during ambient storage. Such behavior is widely discussed in the context of “surface aging” or “hydrophobic recovery”, where adsorption of airborne hydrocarbons and/or re-equilibration of polar sites reduces the apparent surface energy over time, thereby increasing the contact angle [51].

Figure 8 presents mean contact angles with confidence intervals (α = 0.05). The implantation effect is most pronounced for the 5 h dataset, where the relative decrease in average contact angle exceeds 70% compared to the virgin tool (Figure 9). The stronger effect shortly after implantation is consistent with the interpretation that the freshly treated surface contains a higher density of polar/active sites, which progressively become masked or neutralized during storage (e.g., by hydrocarbon adsorption) [51].

From a functional perspective, wettability (and more generally surface energy) can influence interfacial phenomena relevant to tribological contacts. Several studies report correlations between wettability and friction/wear behavior, particularly in the presence of liquids, where improved spreading and interfacial energy balance can modify the friction coefficient [52]. In machining-related contexts, controlling cemented carbide surface wettability has been proposed as a lever to influence tool–workpiece interactions and performance. In the present case, the implantation-induced increase in adhesion tendency (implied by lower water contact angle) may facilitate transfer and retention of species from the machined wood-based material (including carbon-rich fragments), supporting the formation of a secondary layer reported previously for similar systems [7]. Such a layer can act as a tribofilm that reduces shear stresses and contributes to lower wear and longer tool life; however, confirming this mechanism would benefit from complementary surface-chemistry analysis (e.g., XPS/ToF-SIMS) and correlation with tribological or cutting tests.

## 4. Conclusions

Based on the results, the following conclusions can be drawn:

- Nitrogen ion implantation in the tested range of parameters (the acceleration voltage and the ion fluence) does not significantly change the surface roughness of the WC–Co surface. Ion implantation reduces the contact angle by more than 70% for measurements 5 h after the modification. The above effect is not long-lasting, and after 30 h it decreases to 34%.
- With an increase in the implanted dose, the value of the contact angle decreases significantly. A five-times-higher dose results in a more than three-times reduction in the contact angle.
- A decrease in the contact angle indicates improved wettability of the ion-implanted WC–Co surface, which may be beneficial from the point of view of tool–workpiece interfacial interactions during wood machining.

## Figures and Tables

**Table 1:** Values the energy of the nitrogen-implanted ions for the acceleration voltage of 50 kV.

Ion Species	Percentage Charge State Distribution (%)	Acceleration Voltage (kV)	Energy (keV)
N_2_^+^	67	50	25
N^+^	33	50	50

**Table 2:** Values of the parameters of the nitrogen peak and the sputtering yield.

Implanted Ions	Peak Volume Dopant Concentration *N_max_* (cm^−3^)	Projected Range *R_p_* (nm)	Range Straggling Δ*R_p_* (nm)	Skewness	Kurtosis	Sputtering Yield *Y* (Atoms/Ion)
N_2_^+^ + N^+^	2.07 × 10^22^ for 1 × 10^17^ cm^−2^ 1.04 × 10^23^ for 5 × 10^17^ cm^−2^	31.6	37.8	0.8574	3.6237	0.49
ACS	1.99 × 10^22^ for 1 × 10^17^ cm^−2^ 9.95 × 10^22^ for 5 × 10^17^ cm^−2^	31.8	32.4	0.2732	2.5179	0.48

## Data Availability

The original contributions presented in this study are included in the article. Further inquiries can be directed to the corresponding authors.
